# Associations of neutrophil/high-density lipoprotein cholesterol ratio with frailty and its mortality

**DOI:** 10.3389/fendo.2024.1495139

**Published:** 2025-01-06

**Authors:** Jianqiang Zhang

**Affiliations:** ^1^ Department of Medical Intensive Care Unit, The First Affiliated Hospital, and College of Clinical Medicine of Henan University of Science and Technology, Luoyang, China; ^2^ Department of Neurology, The First Affiliated Hospital, and College of Clinical Medicine of Henan University of Science and Technology, Luoyang, China

**Keywords:** neutrophil/high-density lipoprotein cholesterol ratio, frailty, mortality, NHANES, observational study

## Abstract

**Background:**

Frailty is an increasingly important determinant in the field of health, and its identification has important clinical significance in the field of critical care medicine. However, there are still a large number of challenges in quick and accurate identification of frailty. This study aims to evaluate the value of the neutrophil/high-density lipoprotein cholesterol ratio (NHR) in frailty and its long-term survival.

**Methods:**

Adult participants from seven study cycles of the National Health and Nutrition Examination Survey (NHANES) database were included. Frailty was assessed with a 49-item Frailty Index (FI). Weighted logistic regression, restricted cubic spline (RCS), and Cox regression were used to analyze the association of NHR with frailty and its long-term survival. In addition, subgroup and interaction analyses were also performed.

**Results:**

A total of 34,382 adult participants aged 47.6 on average were included, and 16,950 (48.8%) of them were males. After the adjustment of potential confounding variables, an increase of one standard deviation (SD) in NHR resulted in the increase of the incidence of frailty by 11% (OR: 1.11, 95% CI: 1.04-1.18, P = 0.002). RCS showed a J-shaped association between NHR and frailty, which was robust in all subgroups according to the subgroup analysis. In addition, the survival analysis revealed that NHR was significantly positively associated with all-cause (HR: 1.12, 95% CI: 1.07-1.17, P < 0.0001), cardiocerebrovascular disease (CCD)-specific (HR: 1.21, 95% CI: 1.11-1.33, P < 0.0001), and cancer-specific mortality risks (HR: 1.13, 95% CI: 1.07-1.19, P < 0.0001) in frail individuals.

**Conclusion:**

In the American adult population, NHR maintains a J-shaped relationship with frailty. In addition, NHR can help predict long-term mortality in frail individuals. This study demonstrates that NHR may become an effective predictor of frailty and its mortality.

## Introduction

With the aggravation of population aging, the prevalence of frailty is on the rise. Frailty is characterized by a comprehensive decline in the physiological functions of the body and increased susceptibility to stressors (1). Globally, frailty poses a huge challenge to the current medical environment (2). Individuals with physical frailty have increased risk of adverse health outcomes, including but not limited to death, disability, hospitalization, and dementia (3). Studies have shown that frailty can be prevented and its progression can be slowed, and strategies play a crucial role (4). Therefore, in the context of aging today, early identification of frailty enables multidisciplinary teams to formulate potential mitigation plans for early and effective improvement of the quality of life of patients.

It is currently quite complex and difficult to assess and measure frailty in the overall population since it requires the help of dozens of physical or psychological indicators (5). In addition, although the incidence of frailty is high in the elderly population, recent studies have shown that frailty also exists in a considerable number of young people, especially in economically underdeveloped areas (6). Moreover, frailty is a dynamic state and may fluctuate over time, which increases the difficulty and cost of identifying it in clinical practice (7). Inflammation is closely related to frailty and may become a potential marker of frailty. The level of inflammatory markers in the blood is an important sign of chronic diseases, disability, frailty, and premature death (8). There is evidence that the levels of C-reactive proteins (CRPs) and interleukin-6 (IL-6) increase in patients with frailty and pre-frailty (9). In addition to traditional inflammatory markers, some new composite inflammatory markers, such as the systemic immune-inflammation index (SII), systemic inflammation response index (SIRI), neutrophil-to-lymphocyte ratio (NLR), have been confirmed non-linearly related to frailty in the American population (10, 11). Blood lipids may also be involved in the progression of frailty. Studies have shown that malnutrition caused by low levels of high-density lipoprotein cholesterol (HDL-C) may eventually lead to or worsen the state of frailty (12). Importantly, early studies have revealed that simple measurement of HDL-C levels in the frail elderly residing in nursing homes is even helpful to determine the frailty status (13). The neutrophil/high-density lipoprotein cholesterol ratio (NHR) is a composite index combining inflammatory cells and lipid metabolism, and it has been proven significantly related to depression, metabolic syndrome (metS), and the risk of cardiovascular death (14–16). However, the relationship between NHR and frailty remains unclear. In addition, the risk of adverse prognosis always increases in patients with the complication of frailty no matter what type of disease they have. According to Hanlon et al. (6), frailty was significantly positively associated with mortality in the UK Biobank after the adjustment for the number of chronic diseases, sociodemographic factors, and lifestyles. Fan et al. (17) collected the information of more than 500,000 patients from the China Kadoorie Biobank, and found that frailty was associated with all-cause and cause-specific mortality among both young and old Chinese people. An American study involving 5,672 65-year-old participants showed that frailty was associated with increased risk of death, and improvement of frailty symptoms might not reduce the risk (18). Therefore, exploring the relationship between NHR and the risk of death in frail individuals has practical clinical significance.

This study aims to explore the relationship of NHR with frailty and the long-term prognosis (including all-cause, cardiocerebrovascular (CCD)-specific, and cancer-specific mortality) in the American adult population.

## Methods

### Participants

**Figure 1:**
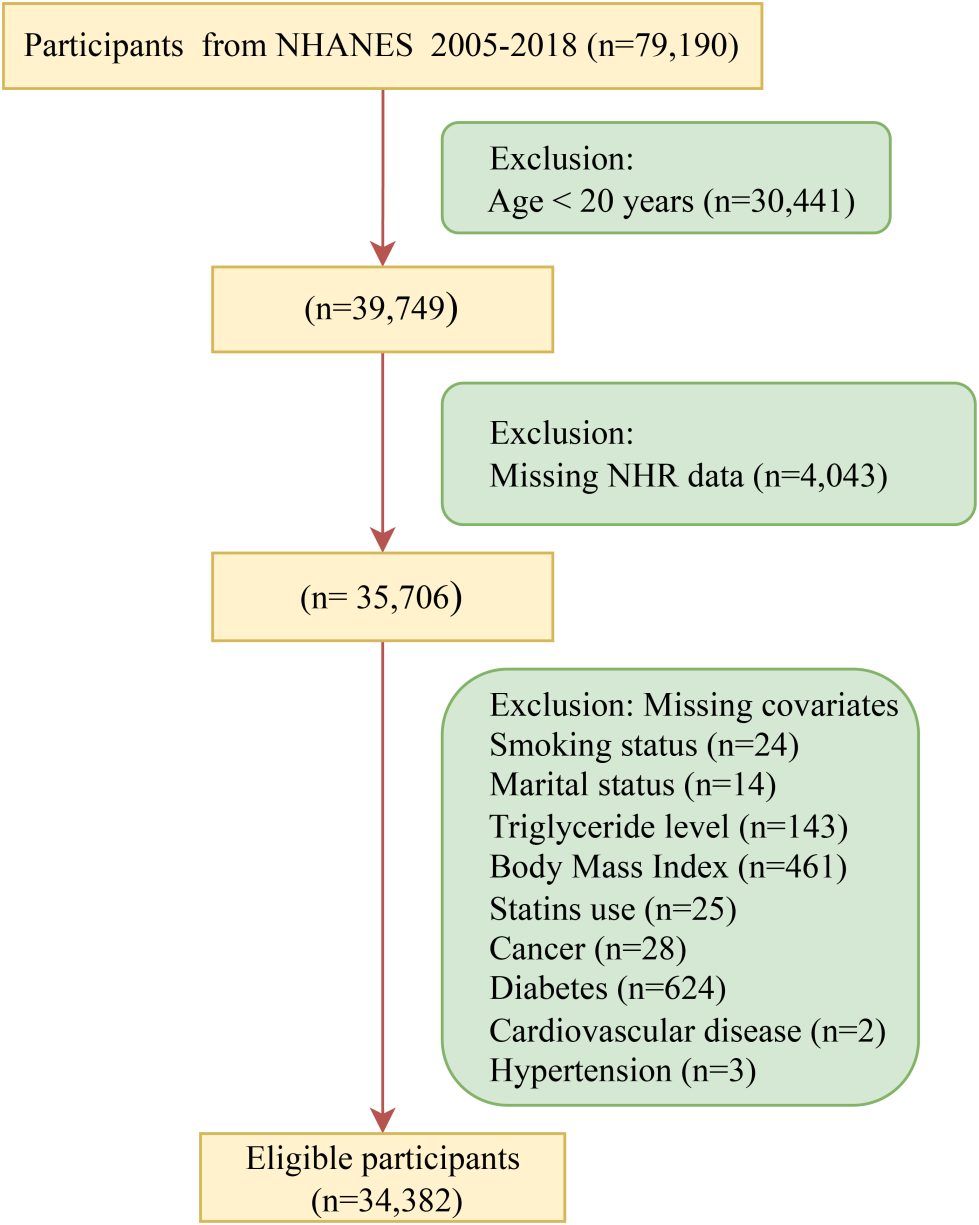
Flow chart of participant recruitment. NHR, neutrophil/high-density lipoprotein cholesterol ratio; NHANES, National Health and Nutrition Examination Survey.

This study initially included participants from seven cycles (2005 to 2018) of the National Health and Nutrition Examination Survey (NHANES) database. After the exclusion of individuals younger than 20 years old (N = 30,441), those with NHR missing (N = 4,043), and those with missing covariates (N = 1,324), a total of 34,382 participants were finally included (**Figure 1**). All participants have signed the written informed consent and agreed to take part in the NHANES research. This research was approved by the Research Ethics Review Committee of the National Center for Health Statistics.

### NHR

NHR is the ratio of the neutrophil count (10^3^ cells/μL) to the HDL-C concentration (mmol/L) (15). Both the neutrophil count and HDL-C concentration were obtained through laboratory tests. NHR had a skewed distribution, so it was standardized in this study. Specifically, NHR was transformed into a distribution with a mean of 0 and a standard deviation (SD) of 1 by the Z-score method.

**Figure 2:**
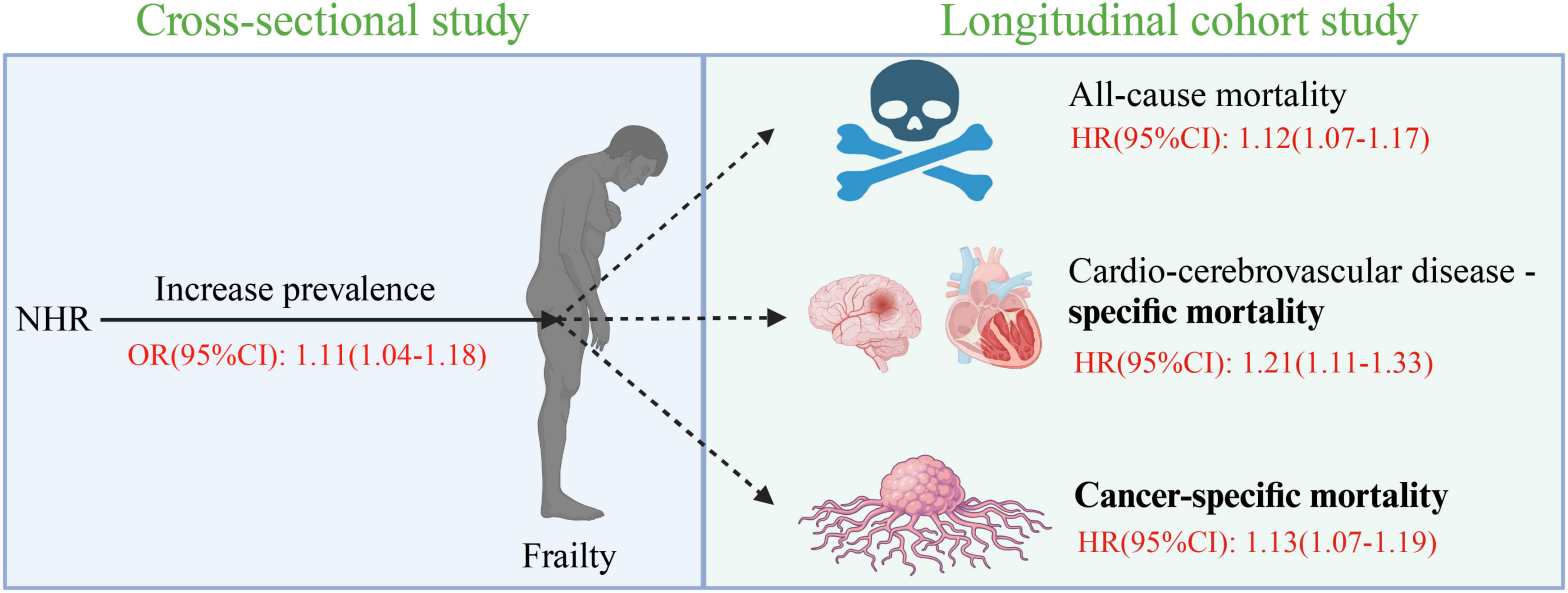
Figure abstract. NHR, neutrophil/high-density lipoprotein cholesterol ratio; OR, odds ratio; HR, hazard ratio; CI, confidence interval.

### Diagnosis of frailty

In this study, frailty was diagnosed in accordance with the diagnostic criteria proposed by Hakeem et al. (10, 19) Specifically, a 49-item Frailty Index (FI) was used for frailty diagnosis. This scale includes 49 items of multiple dimensions, comprehensively considering the cognitive level, physical skills, daily activity level, depressive symptoms, physical health status, chronic disease conditions, laboratory test indicators, and healthcare status. Details of this assessment scale can be found in **Supplementary Table 1**. The score of each item ranges from 0 to 1. The final frailty score was calculated by dividing the sum the scores of specific defective items by the total score of all items (20). According to previous studies, the cut-off value of the FI was 0.21. Thus, an FI score of ≥ 0.21 was defined as frailty, while a FI score of < 0.21 was defined as non-frailty (21).

### Survival outcome

Death certificate data were derived from the National Death Index (NDI). NHANES and NDI data were linked in a probabilistic matching manner to obtain the survival of the participants, and a series of identification symbols were used. Participants who failed the NDI matching were assumed to be alive by default (22, 23). The follow-up duration was calculated in years, which refers to the time interval between the start of the follow-up to the date of death or December 31, 2019. Disease-specific mortality was determined according to the guidelines of the Tenth Revision of the International Statistical Classification of Diseases (ICD-10). Cancer-specific mortality is the number of deaths caused by malignant neoplasms (C00-C97) (24). CCD-specific mortality refers to the number of deaths caused by cardiovascular diseases (CVDs) (I00-I09, I11, I13, I20-I51) and cerebrovascular diseases (I60-I69) (25).

### Covariates

Based on existing publications and clinical practice, demographic factors, lifestyles, a physical indicator, medication usage, comorbidities, and lipid levels that might affect both the prevalence of frailty and the NHR level were included for analysis in this study. Demographic factors include age, sex, race/ethnicity, education attainment, the marital condition, and the economic status from the NHANES database. Lifestyles mainly include smoking, alcohol consumption, weekly exercise intensity, and daily dietary energy. The physical indicator is body mass index (BMI). Medication usage refers to lipid-lowering drugs. The analyzed comorbidities were cancer, hypertension, diabetes mellitus (DM), and CVDs. Lipids included total blood cholesterol and triglyceride levels. The CVDs reported by the participants themselves include coronary artery disease, congestive heart failure, myocardial infarction, stroke, and angina pectoris. Hypertension was diagnosed if an individual met any one or more than one of the following three criteria (26). (1) The average systolic blood pressure was ≥ 140 mmHg or the diastolic blood pressure was ≥ 90 mmHg. (2) Hypertension was diagnosed by a doctor. (3) Antihypertensive medications prescribed by a doctor were taken. Individuals meeting one or more than one of the following five criteria were considered to have DM (27). (1) The fasting plasma glucose level was ≥ 7.0 mmol/L or the blood glucose level for a 2-hour oral glucose tolerance test was ≥ 11.1 mmol/L. (2) The random blood glucose level was ≥ 11.1 mmol/L. (3) The glycated hemoglobin level was ≥ 6.5%. (4) Diabetes medications or insulin were taken. (5) Diabetes was diagnosed by a doctor.

### Statistical analysis

NHANES conducts research using a complex sampling method to obtain a sample representative of all residents of the United States. The sampling procedure consists of four stages, which are counties, segments, households, and individuals. In this study, participants from seven cycles were included, so a laboratory weighting of 1 in 7 was used for data analysis. The t-test or Wilcoxon rank-sum test was used to analyze continuous variables. For categorical variables, the chi-square (χ^2^) test was used to compare baseline characteristics between the frail and healthy control groups. Four multivariate logistic or Cox regression models were employed to estimate the association of NHR with frailty and its survival. Model 0 was not adjusted for any confounding factors. Model 1 was adjusted for only demographic factors. Model 2 was also adjusted for lifestyles and BMI in addition to demographic factors. Model 3 was adjusted for all the confounding factors, including demographic factors, lifestyles, BMI, medication history, comorbidities and blood lipid levels.

In logistic and Cox regression models, NHR was used as a continuous or categorical variable based on quartiles. Restricted cubic spline (RCS) is a reliable method for analyzing nonlinear associations. In this study, a RCS was fitted with 3-knots based on weighted logistic or Cox regression. Both logistic and Cox regression models were subjected to collinearity diagnosis. The Schoenfeld residual method was also used to test whether the Cox regression model satisfied the proportional hazards assumption. In addition, the subgroup analysis and likelihood ratio test were also performed to identify potentially vulnerable populations.

The sensitivity analysis was conducted. Firstly, according to the definition of frailty by Blodgett et al. (28), patients with an FI score of ≤ 0.10 were non-frail, those with an FI score of 0.10 < FI ≤ 0.21 were vulnerable, those with an FI score of 0.21 < FI ≤ 0.45 were frail, and those with an FI score of > 0.45 were most frail. The association between NHR and outcomes was explored based on this criterion. Secondly, three additional covariates were adjusted for, including the use of statins, fibrates, and the combination of statins and ezetimibe. The statins were classified into low, moderate, and high intensity according to their ability to reduce low-density lipoprotein cholesterol (LDL-C). Thirdly, patients were divided into low- and high-NHR groups according to the median value of NHR.

All statistical procedures were implemented in R language. “NhanesR”, “tidyverse”, “rio”, and “data.table” packages were used for data cleaning. “Survival”, “survey”, and “rms” packages were employed to fit regression models. “Car” and “survival” packages were used for collinearity diagnosis and proportional hazards assumption testing. In this study, a two-tailed P value of < 0.05 was considered statistically significant.

## Results

### Population characteristics

As shown in **Figure 1**, after sequentially excluding individuals younger than 20 years old and those with missing exposure variables and covariates, a total of 34,382 participants were included in this study. **Table 1** presents the characteristics of the participants. The participants had an average age of 47.6, and 16,950 (48.8%) were men. Among the 34,382 participants, 7,420 were diagnosed with frailty. Compared with the non-frail group, the frail group had a higher NHR and lower levels of total blood cholesterol, LDL-C, and HDL-C. The levels of triglycerides, Castelli risk index I and atherogenic index of plasma were also higher in the frail group. In terms of demographics, the frail group had an older age, a higher proportion of females and non-Hispanic blacks, and a lower proportion of advanced educational attainment. The ratio of divorced or widowed individuals in the frail group more than doubled, compared with that in the non-frail group. Moreover, there were more impoverished individuals in the frail group. In terms of lifestyles, more people in the frail group had a history of smoking and drinking, did less weekly exercise, and had lower daily dietary energy. Furthermore, in the frail group, weight management was poor, and more people used statins (including low-, moderate-, and high-intensity), fibrates, and the combination of statins and ezetimibe. Finally, the prevalence of cancer, diabetes, hypertension, and CVD in the frail group was at least twice as high as that in the non-frail group.

**Table 1:** Weighted baseline characterization for cross-sectional study.

Characteristics	Total (N=34,382)	Control (N=26,962)	Frailty (N=7,420)	*P*-value
NHR, Median (IQR)	3.06 (2.15,4.31)	2.99 (2.11,4.19)	3.49 (2.38,4.92)	< 0.0001
Age (year), Mean (S.E.)	47.60 (0.23)	45.53 (0.23)	57.70 (0.32)	< 0.0001
Total cholesterol level (mmol/L), Mean (S.E.)	5.01 (0.01)	5.03 (0.01)	4.93 (0.02)	< 0.0001
Triglyceride level (mmol/L), Mean (S.E.)	1.72 (0.01)	1.68 (0.01)	1.96 (0.03)	< 0.0001
Low-density lipoprotein cholesterol, Mean (S.E.)	2.95 (0.01)	2.97 (0.01)	2.82 (0.02)	< 0.0001
High-density lipoprotein cholesterol, Mean (S.E.)	1.38 (0.01)	1.39 (0.01)	1.33 (0.01)	< 0.0001
Castelli risk index I, Mean (S.E.)	3.92 (0.01)	3.90 (0.02)	4.01 (0.03)	< 0.0001
Castelli risk index II, Mean (S.E.)	2.26 (0.01)	2.26 (0.01)	2.26 (0.03)	0.99
Atherogenic index (AI), Mean (S.E.)	2.92 (0.01)	2.90 (0.02)	3.01 (0.03)	< 0.0001
Atherogenic index of plasma (AIP), Mean (S.E.)	-0.06 (0.00)	-0.08 (0.00)	0.03 (0.01)	< 0.0001
Sex, n (%)				< 0.0001
Female	17,432 (51.2)	13,073 (49.0)	4,359 (62.0)	
Male	16,950 (48.8)	13,889 (51.0)	3,061 (38.0)	
Race/Ethnicity, n (%)				< 0.0001
Mexican American	5,422 (8.5)	4,448 (8.9)	974 (6.7)	
Non-Hispanic Black	7,122 (10.6)	5,215 (9.7)	1,907 (15.3)	
Non-Hispanic White	14623 (67.6)	11,380 (68.0)	3243 (65.6)	
Other Hispanic	3,338 (5.6)	2,624 (5.6)	714 (5.4)	
Other Race - Including Multi-Racial	3,877 (7.7)	3,295 (7.8)	582 (7.1)	
Educational level, n (%)				< 0.0001
No college	16,435 (39.3)	12,004 (36.5)	4,431 (52.6)	
or equivalent	17,947 (60.8)	14,958 (63.5)	2,989 (47.5)	
Marital status, n (%)				< 0.0001
No married	6,125 (17.6)	5,216 (18.9)	909 (11.3)	
Divorced or separated or widowed	7,672 (18.5)	4,930 (15.5)	2,742 (33.4)	
Already married or cohabitation	20,585 (63.8)	16,816 (65.6)	3,769 (55.3)	
PIR, n (%)				< 0.0001
<1.3	98,44 (19.6)	6,876 (17.1)	2,968 (31.5)	
1.3–3.5	11,869 (33.2)	9,312 (32.5)	2,557 (36.5)	
>3.5	9,665 (40.2)	8,483 (43.5)	1,182 (24.2)	
Not report	3,004 (7.0)	2,291 (6.8)	713 (7.8)	
Drinking status, n (%)				< 0.0001
Never drinker	4,348 (9.8)	3,307 (9.5)	1,041 (11.2)	
Former drinker	5,004 (12.1)	3,228 (10.0)	1,776 (22.1)	
Current drinker	21,263 (68.7)	17,625 (71.5)	3,638 (54.9)	
Not report	3,767 (9.5)	2,802 (9.0)	965 (11.9)	
Smoking status, n (%)				< 0.0001
Never smoked	18,990 (55.0)	15,737 (57.7)	3,253 (42.1)	
Former smoker	8,312 (24.7)	5,984 (23.4)	2,328 (31.1)	
Current smoker	7,080 (20.3)	5,241 (18.9)	1,839 (26.7)	
Physical activity (MET, minutes/week, n (%)				< 0.0001
<700	6,586 (19.5)	5,194 (19.6)	1,392 (19.3)	
700-2400	7,572 (23.9)	6,364 (25.3)	1,208 (17.3)	
>=2400	11,188 (35.0)	9,679 (37.6)	1,509 (22.1)	
Not report	9,036 (21.6)	5,725 (17.6)	3,311 (41.3)	
Energy intake (kcal/day), n (%)				< 0.0001
Low	14,311 (38.6)	10,616 (36.8)	3,695 (47.4)	
High	17,923 (56.3)	14,759 (58.5)	3,164 (45.8)	
Not report	2,148 (5.1)	1,587 (4.7)	561 (6.8)	
Body mass index, n (%)				< 0.0001
<25 kg/m^2^	9,952 (30.1)	8,521 (32.4)	1,431 (18.8)	
>=25 kg/m^2^	24,430 (69.9)	18,441 (67.6)	5,989 (81.2)	
Statins use, n (%)				< 0.0001
No	27,820 (83.0)	23,294 (87.2)	4,526 (62.5)	
Yes	65,62 (17.0)	3,668 (12.8)	2,894 (37.5)	
Statins categories, n (%)				< 0.0001
Low intensity	14 (0.1)	9 (0.0)	5 (0.2)	
Moderate intensity	3,722 (9.5)	2,090 (7.1)	1,632 (21.2)	
High intensity	2,826 (7.4)	1,569 (5.7)	1,257 (16.0)	
Not use	27,820 (83.0)	23,294 (87.2)	4,526 (62.5)	
Statins combine Ezetimibe, n (%)				< 0.0001
No	34,019 (99.0)	26,765 (99.3)	7,254 (97.8)	
Yes	363 (1.0)	197 (0.7)	166 (2.2)	
Fibrates use, n (%)				< 0.0001
No	34,061 (99.1)	26,810 (99.4)	7,251 (97.5)	
Yes	321 (1.0)	152 (0.6)	169 (2.6)	
Cancer, n (%)				< 0.0001
No	31,172 (90.1)	25,128 (92.3)	6,044 (79.2)	
Yes	3,210 (9.9)	1,834 (7.7)	1,376 (20.8)	
DM, n (%)				< 0.0001
DM	6,513 (14.1)	3,331 (9.2)	3,182 (38.1)	
IFG	1,640 (5.0)	1,286 (4.9)	354 (5.6)	
IGT	1,281 (3.4)	1,030 (3.4)	251 (3.4)	
No	24,948 (77.5)	21,315 (82.5)	3,633 (53.0)	
CVD, n (%)				< 0.0001
No	30,573 (91.3)	25,701 (96.0)	4,872 (68.2)	
Yes	3,809 (8.8)	1,261 (4.0)	2,548 (31.8)	
Hypertension, n (%)				< 0.0001
No	19,758 (62.3)	17,763 (69.1)	1,995 (29.3)	
Yes	14,624 (37.7)	9,199 (30.9)	5,425 (70.8)	

NHR, neutrophil/high-density lipoprotein cholesterol ratio; IQR, Interquartile Range; SE, standard error; PIR, poverty-to-income ratio; MET, metabolic equivalent; DM, diabetes mellitus; IFG, impaired fasting glycaemia; IGT, impaired glucose tolerance; CVD, cardiovascular diseases.

A total of 7,415 frail participants were included in the survival analysis after excluding 5 individuals who were lost to follow-up. During the average follow-up period of 6.5 years, 1,806 participants died for various reasons, including CCD (593 deaths) and cancer (325 deaths). The baseline characteristics of the death and survivor groups are shown in **Supplementary Table 2**. Specifically, the NHR was larger and the total blood cholesterol and triglyceride levels were lower in the death group. The death group had an older age, a higher proportion of men and non-Hispanic whites, and a lower proportion of a higher education degree. There were more divorced or widowed and impoverished individuals in the death group. In addition, more people in the death group had smoked and drunk before. They did less weekly exercise and had lower daily dietary energy, so the proportion of overweight and obesity was lower than that in the survivor group. Finally, the proportion of using statins in the death group was relatively higher, and the prevalence rates of cancer, diabetes, hypertension, and CVD were all increased compared with those in the survivor group.

### The association of NHR with frailty

**Table 2** shows the results of weighted logistic regression of NHR and frailty. When NHR was included as a continuous variable, Models 0-3 all showed a steady increase in the prevalence of frailty. In Model 3, every 1 SD increase of NHR led to an increase of the prevalence of frailty by 11% (OR: 1.11, 95% CI: 1.04 - 1.18, P = 0.002). After NHR was included as a four-category variable in Model 3 with all confounding factors adjusted for, the odds of frailty in Quartile 2-4 groups were not significantly different from those in the Quartile 1 group (all P > 0.05).

**Table 2:** OR estimates for the association between NHR and frailty.

		Model 0		Model 1		Model 2		Model 3	
		OR (95%CI)	*P-*value	OR (95% CI)	*P-*value	OR (95%CI)	*P-*value	OR (95%CI)	*P-*value
Frailty ~NHR	Per SD	1.34 (1.28,1.40)	<0.0001	1.53 (1.45, 1.61)	<0.0001	1.34 (1.27, 1.41)	<0.0001	1.11 (1.04, 1.18)	0.002
	Quartile 1	reference		reference		reference		reference	
	Quartile 2	1.14 (1.01,1.27)	0.03	1.29 (1.14, 1.46)	<0.0001	1.13 (1.00, 1.27)	0.06	0.97 (0.84, 1.11)	0.62
	Quartile 3	1.33 (1.17,1.51)	<0.0001	1.66 (1.43, 1.92)	<0.0001	1.32 (1.13, 1.53)	<0.001	0.98 (0.83, 1.15)	0.80
	Quartile 4	1.87 (1.67,2.10)	<0.0001	2.67 (2.33, 3.07)	<0.0001	1.86 (1.63, 2.14)	<0.0001	1.12 (0.95, 1.32)	0.16
	*P* for trend		<0.0001		<0.0001		<0.0001		0.13
	*<*3.19	1.03 (0.98,1.09)	0.25	1.10 (1.04, 1.17)	0.002	1.00 (0.94, 1.06)	0.99	0.87 (0.80, 0.93)	<0.001
	≥3.19	1.34 (1.25,1.43)	<0.0001	1.50 (1.39,1.62)	<0.0001	1.38 (1.27,1.49)	<0.0001	1.22 (1.12,1.34)	<0.0001

Model 0: Crude model. Model 1: Adjusted for age, sex, race, marital status, education, and poverty-income ratio. Model 2: Additionally adjusted for drinking, smoking, total energy intake, weekly physical activity level, and BMI. Model 3: Additionally, adjusted for diabetes, cancer, hypertension, CVD, blood cholesterol levels, blood triglyceride levels, lipid-lowering drugs. NHR, neutrophil/high-density lipoprotein cholesterol ratio; CVD, cardiovascular disease; SD, standard deviation; BMI, body mass index; OR, odds ratio; CI, confidence interval.

**Figure 3:**
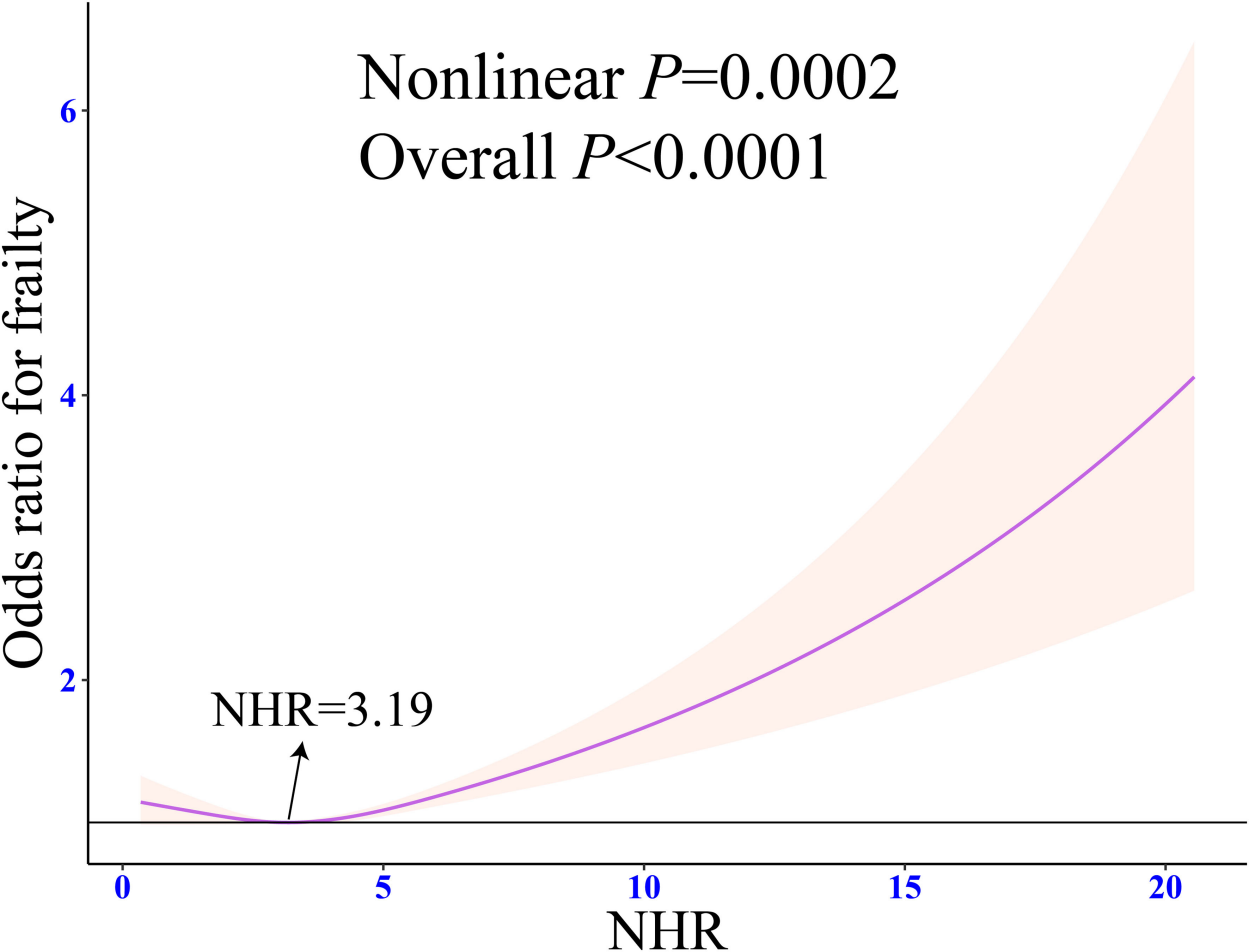
Weighted restricted cubic spline regression of NHR with frailty. The adjusted restricted triple spline model was adjusted for age, sex, race, marital status, education, poverty-to-income ratio, drinking, smoking, total energy intake, weekly physical activity level, BMI, diabetes, cancer, hypertension, CVD, blood cholesterol levels, blood triglyceride levels, lipid-lowering drugs. NHR, neutrophil/high-density lipoprotein cholesterol ratio; BMI, body mass index; CVD, cardiovascular diseases; OR, odds ratio; CI, confidence interval.

RCS regression showed a significant J-shaped association between NHR and frailty (Nonlinear P = 0.0002). When NHR was 3.19, the prevalence of frailty was the lowest (**Figure 3**). Piecewise logistic regression revealed that after the adjustment for all the potential confounding variables (Model 3), the odds of frailty had a significant negative association with NHR when it was < 3.19 (OR: 0.87, 95% CI: 0.80 - 0.93, P < 0.001). An NHR of ≥ 3.19 was significantly positively associated with the prevalence of frailty (OR: 1.22, 95% CI: 1.12 - 1.34, P < 0.0001) (**Table 2**).

**Figure 4:**
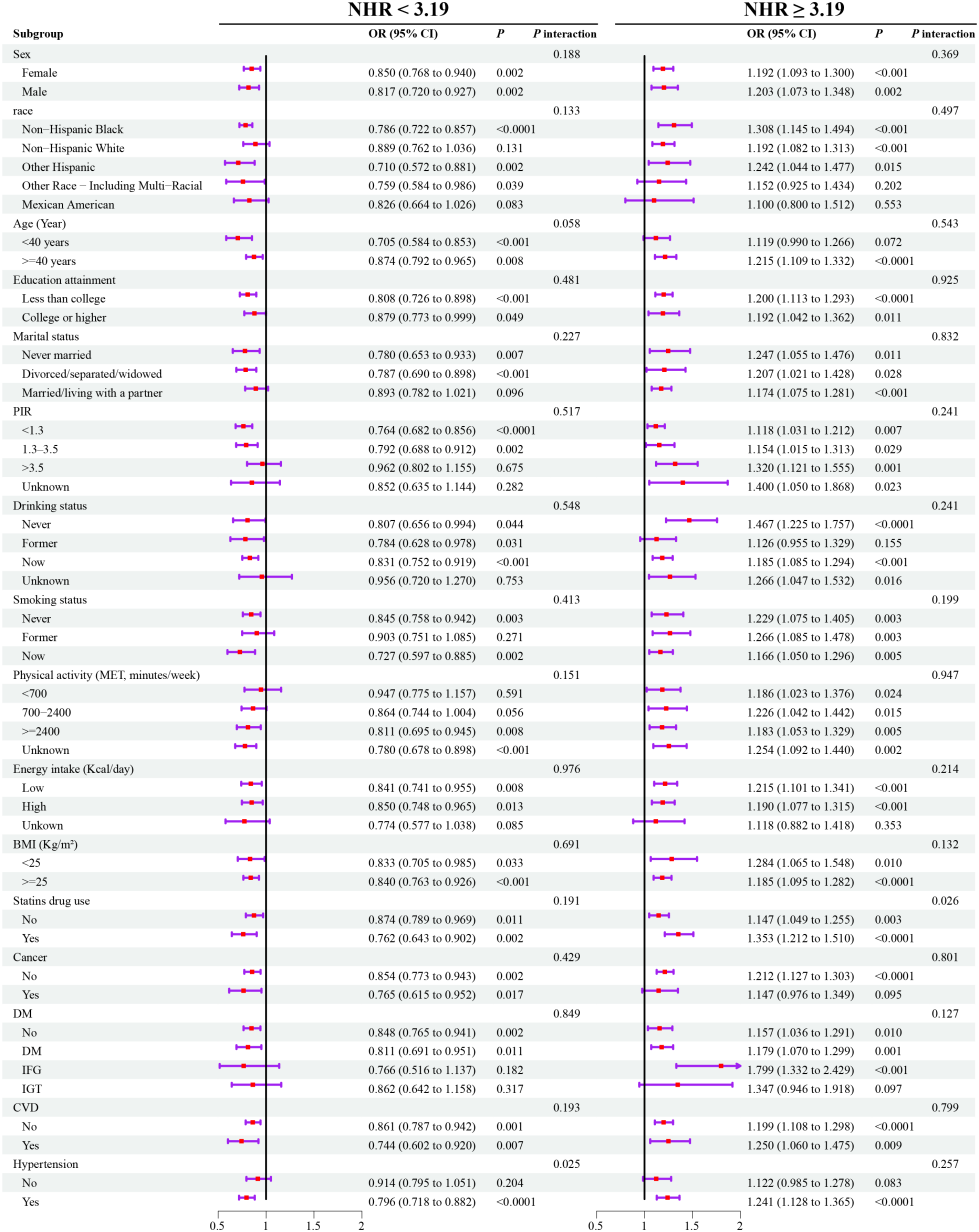
Weighted subgroup analyses for the association between NHR and frailty. Models were adjusted for age, sex, race, marital status, education, poverty-to-income ratio, drinking, smoking, total energy intake, weekly physical activity level, BMI, diabetes, cancer, hypertension, CVD, blood cholesterol levels, blood triglyceride levels, lipid-lowering drugs. OR, odds ratio; NHR, neutrophil/high-density lipoprotein cholesterol ratio; BMI, body mass index; CVD, cardiovascular diseases; PIR, poverty-to-income ratio; MET, metabolic equivalent; DM, diabetes mellitus; IFG, impaired fasting glycaemia; IGT, impaired glucose tolerance; OR, odds ratio; CI, confidence interval.

The subgroup analysis showed that the basic association between NHR and frailty was consistent among different populations (all P for interaction > 0.05) (**Figure 4**).

### The relationship of NHR with all-cause and cause-specific mortality among patients with frailty

In the weighted Cox regression Model 3, per 1 SD increase in NHR resulted in the increase of the all-cause, CCD-specific and cancer-specific mortality risks by 12% (HR: 1.12, 95% CI: 1.07 - 1.17, P < 0.0001), 21% (HR: 1.21, 95% CI: 1.11 - 1.33, P < 0.0001), and 13% (HR: 1.13, 95% CI: 1.07 - 1.19, P < 0.0001), respectively (**Table 3**). When NHR was included as a four-category variable in Model 3, both the all-cause (HR: 1.26, 95% CI: 1.06 - 1.49, P = 0.01) and CCD -specific mortality (HR: 1.55, 95% CI: 1.12 - 2.13, P = 0.01) in the highest NHR quartile group was significantly higher than that in the lowest NHR quartile group. However, there was no difference in the cancer-specific mortality among the four NHR groups (all P > 0.05).

**Table 3:** HR estimates for the association between NHR and all-cause and cause-specific mortality in patients with frailty.

		Model 0		Model 1		Model 2		Model 3		
		HR (95%CI)	*P-*value	HR (95%CI)	*P-*value	HR (95%CI)	*P-*value	HR (95%CI)	*P-*value	
All-cause mortality~NHR	Continuous	1.12 (1.07,1.18)	<0.0001	1.15 (1.10,1.20)	<0.0001	1.13 (1.08,1.18)	<0.0001	1.12 (1.07,1.17)	<0.0001
	Quartile 1	ref		ref		ref		ref	
	Quartile 2	1.18 (1.00,1.41)	0.05	0.92 (0.77,1.10)	0.35	0.91 (0.76,1.08)	0.28	0.89 (0.75, 1.06)	0.19
	Quartile 3	1.19 (1.01,1.40)	0.04	1.02 (0.86,1.22)	0.79	1.02 (0.84,1.23)	0.87	0.97 (0.80, 1.18)	0.78
	Quartile 4	1.37 (1.18,1.59)	<0.0001	1.39 (1.19,1.63)	<0.0001	1.33 (1.12,1.57)	<0.001	1.26 (1.06, 1.49)	0.01
	*P* for trend		<0.001		<0.0001		<0.001		0.01
CCD mortality~NHR	Continuous	1.12 (1.04,1.20)	0.002	1.24 (1.14,1.34)	<0.0001	1.24 (1.13,1.35)	<0.0001	1.21 (1.11,1.33)	<0.0001
	Quartile 1	ref		ref		ref		ref	
	Quartile 2	1.47 (1.07,2.03)	0.02	1.05 (0.75,1.48)	0.76	1.04 (0.74,1.45)	0.82	0.99 (0.70,1.40)	0.97
	Quartile 3	1.69 (1.29,2.22)	<0.001	1.41 (1.05,1.89)	0.02	1.40 (1.02,1.92)	0.04	1.34 (0.95,1.89)	0.10
	Quartile 4	1.60 (1.24,2.07)	<0.001	1.63 (1.24,2.14)	<0.001	1.63 (1.21,2.20)	0.001	1.55 (1.12,2.13)	0.01
	*P* for trend		<0.001		<0.0001		<0.001		0.002
Cancer mortality~NHR	Continuous	1.17 (1.05,1.30)	0.004	1.15 (1.10, 1.21)	<0.0001	1.12 (1.06, 1.18)	<0.0001	1.13 (1.07, 1.19)	<0.0001
	Quartile 1	ref		ref		ref		ref	
	Quartile 2	1.13 (0.75,1.69)	0.56	0.84 (0.54, 1.29)	0.42	0.80 (0.52, 1.23)	0.30	0.82 (0.54, 1.24)	0.34
	Quartile 3	0.88 (0.59,1.32)	0.53	0.72 (0.46, 1.12)	0.14	0.71 (0.45, 1.10)	0.12	0.72 (0.46, 1.13)	0.16
	Quartile 4	1.39 (0.95,2.03)	0.09	1.35 (0.85, 2.14)	0.21	1.20 (0.75, 1.93)	0.45	1.27 (0.77, 2.08)	0.35
	*P* for trend		0.21		0.25		0.46		0.38
	<2.58	0.89 (0.69,1.14)	0.34	0.77 (0.59, 1.00)	0.05	0.75 (0.58, 0.97)	0.03	0.71 (0.55, 0.92)	0.01
	≥2.58	1.19 (1.08,1.30)	<0.001	1.17 (1.12,1.23)	<0.0001	1.15 (1.09,1.20)	<0.0001	1.16 (1.10,1.22)	<0.0001

Model 0: Crude model. Model 1: Adjusted for age, sex, race, marital status, education, and poverty-income ratio. Model 2: Additionally adjusted for drinking, smoking, total energy intake, weekly physical activity level, and BMI. Model 3: Additionally, adjusted for diabetes, cancer, hypertension, CVD, blood cholesterol levels, blood triglyceride levels, lipid-lowering drugs. NHR, neutrophil/high-density lipoprotein cholesterol ratio; CVD, cardiovascular disease; CCD, cardiocerebrovascular disease; SD, standard deviation; BMI, body mass index; HR, hazard ratio; CI, confidence interval.

**Figure 5:**
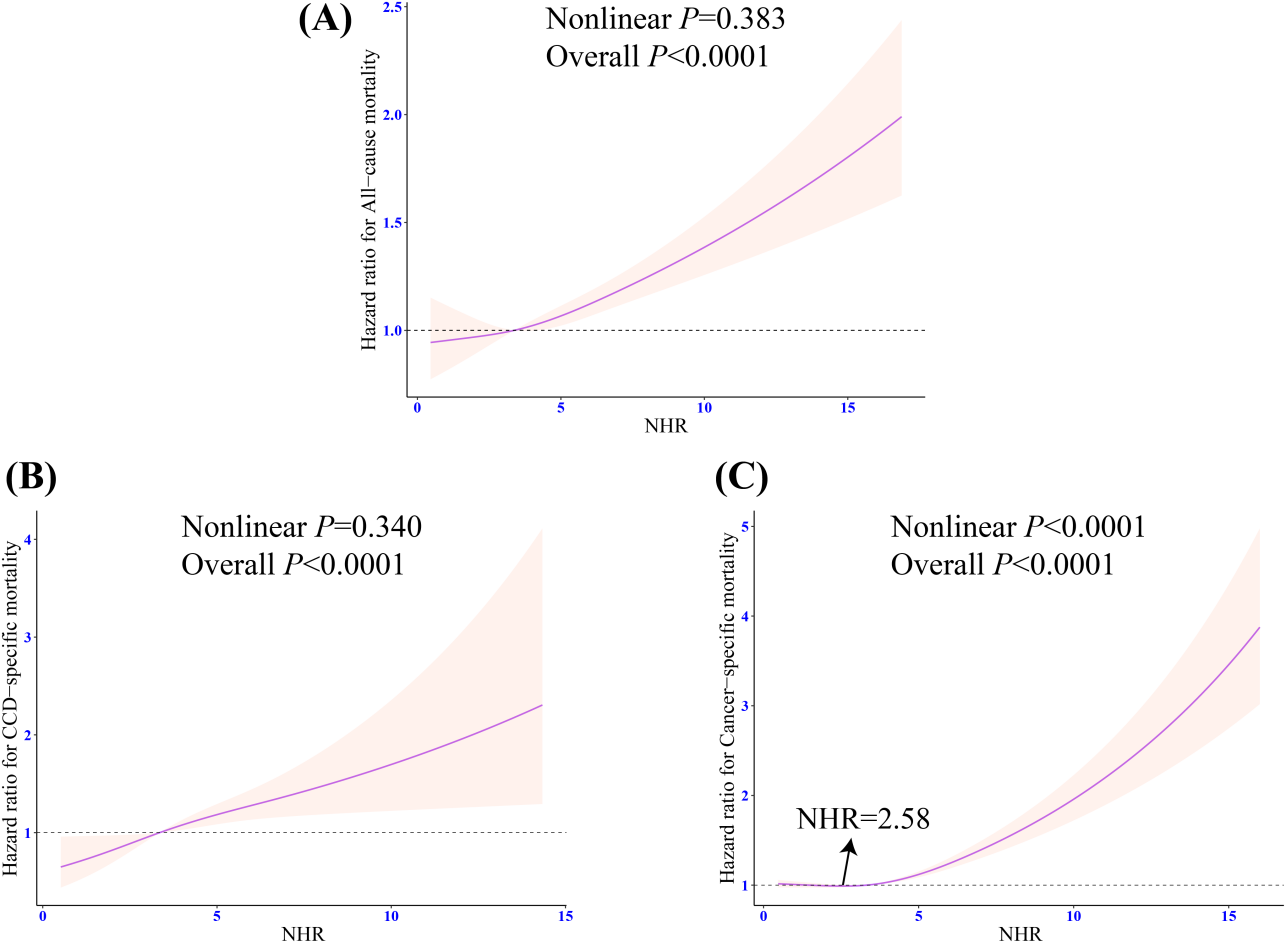
Weighted restricted cubic spline regression of NHR with all-cause and cause-specific mortality in patients with frailty. **(A)** Dose response relationship between NHR and the risk of all-cause mortality; **(B)** Dose response relationship between NHR and the risk of CCD-specific mortality; **(C)** Dose response relationship between NHR and the risk of cancer-specific mortality. The adjusted restricted triple spline model was adjusted for age, sex, race, marital status, education, poverty-to-income ratio, drinking, smoking, total energy intake, weekly physical activity level, BMI, diabetes, cancer, hypertension, CVD, blood cholesterol levels, blood triglyceride levels, lipid-lowering drug. NHR, neutrophil/high-density lipoprotein cholesterol ratio; BMI, body mass index; CVD, cardiovascular diseases; CCD, cardiocerebrovascular disease; HR, hazard ratio; CI, confidence interval.

RCS indicated that there was a linear relationship between NHR and risks of all-cause and CCD-specific mortality (both nonlinear P > 0.05) (**Figures 5A, B**). However, NHR and cancer-specific mortality showed a nonlinear association (nonlinear P < 0.0001), and the inflection point was NHR = 2.58 (**Figure 5C**). Further piecewise Cox regression showed that in Model 3 with all potential confounding variables adjusted for, an NHR value of < 2.58 was significantly negatively associated with the cancer-specific mortality risk (HR: 0.71, 95% CI: 0.55 - 0.92, P = 0.01). An NHR value of ≥ 2.58 increased the risk of cancer-specific mortality (HR: 1.16, 95% CI: 1.10 - 1.22, P < 0.0001) (**Table 3**).

### Sensitivity analysis

The frailty status was classified into four categories in the sensitivity analysis (**Supplementary Table 3**). After the adjustment for all confounding factors (Model 3), NHR was significantly positively associated with vulnerable (OR: 1.05, 95% CI: 1.01 – 1.10, P = 0.03), frail (OR: 1.12, 95% CI: 1.03 – 1.23, P = 0.01), and most frail states in patients (OR: 1.31, 95% CI: 1.08 – 1.60, P = 0.01). As shown in **Supplementary Table 4**, after the adjustment for drug-related covariates, the strength of the association between NHR and frailty remained stable (OR: 1.11, 95% CI: 1.04 – 1.19, P = 0.001). Moreover, NHR remained positively associated with several mortality risks. It can be seen from **Supplementary Figure 1** that the cumulative all-cause and CCD-specific mortality risks of the low- and high-NHR groups differed significantly (P = 0.008 and P = 0.01, respectively). However, there was no significant difference in cancer-specific mortality between the high- and low-NHR groups (P = 0.67). According to **Supplementary Table 5**, compared with the low-NHR group, the high-NHR group had significantly higher all-cause (HR: 1.17, 95% CI: 1.01 – 1.36, P = 0.04) and CCD-specific mortality (HR: 1.43, 95% CI: 1.11 – 1.84, P = 0.01). However, the dichotomized NHR was not related to the risk of cancer-specific mortality (HR: 1.07, 95% CI: 0.75 – 1.51, P = 0.71).

## Discussion

In this large cross-sectional cohort study involving 34,382 participants, the relationship of NHR with frailty and the risk of death was investigated. It was found that NHR was significantly positively associated with the prevalence of frailty after the inflection point at 3.19, but NHR was significantly negatively associated with frailty before this inflection point. The subgroup analysis showed that the association between NHR and frailty was consistent in all subgroups. On the other hand, a greater NHR related to a higher risk of all-cause, CCD-specific, and cancer-specific mortality in 7,415 frail participants with an average follow-up duration of 6.51 years. In conclusion, the results of this study suggest that NHR is an effective evaluation index for frailty and its three types of death risks (**Figure 2**).

It is difficult to identify frailty in the early stage since it may be accompanied by different disease states, which may cause greater consumption of medical resources and worse outcomes. Therefore, it is of great value to explore convenient indicators for fast frailty assessment. Some studies have achieved exciting results. Fox instance, the research by Zhang et al. (10) showed that SII and SIRI had a nonlinear J-shaped association with frailty. However, they excluded participants under 40 years old, which possibly limited the generalizability of the association between inflammation and frailty. The present study showed that the association between NHR and frailty was robust in both the 20-40-year-old and over 40-year-old subgroups. In addition, although Zhang et al. detected the potential nonlinearity, they did not further explore the characteristics of associations between inflammation indexes based on inflection points and frailty. Tang et al. (11) explored the association of inflammation markers derived from whole blood cells with frailty and death in middle-aged and elderly people. They found that NLR, monocyte to lymphocyte ratio (MLR), platelet to lymphocyte ratio (PLR), SII, and SIRI were positively associated with the risk of frailty. Increased NLR, MLR, PLR, SII, and SIRI levels meant a higher risk of death. However, Tang et al. seemed to explain the nonlinear association of the discovered inflammatory indicators with frailty and the risk of all-cause mortality as a simple linear relationship. In addition, although the relationship between age and frailty was relatively clear, frailty also exists in a considerable proportion of young people, especially in economically underdeveloped areas (6). However, the above-mentioned two studies excluded people under 40 and 45 years old, respectively. Thus, their conclusions are not general.

**Figure 6:**
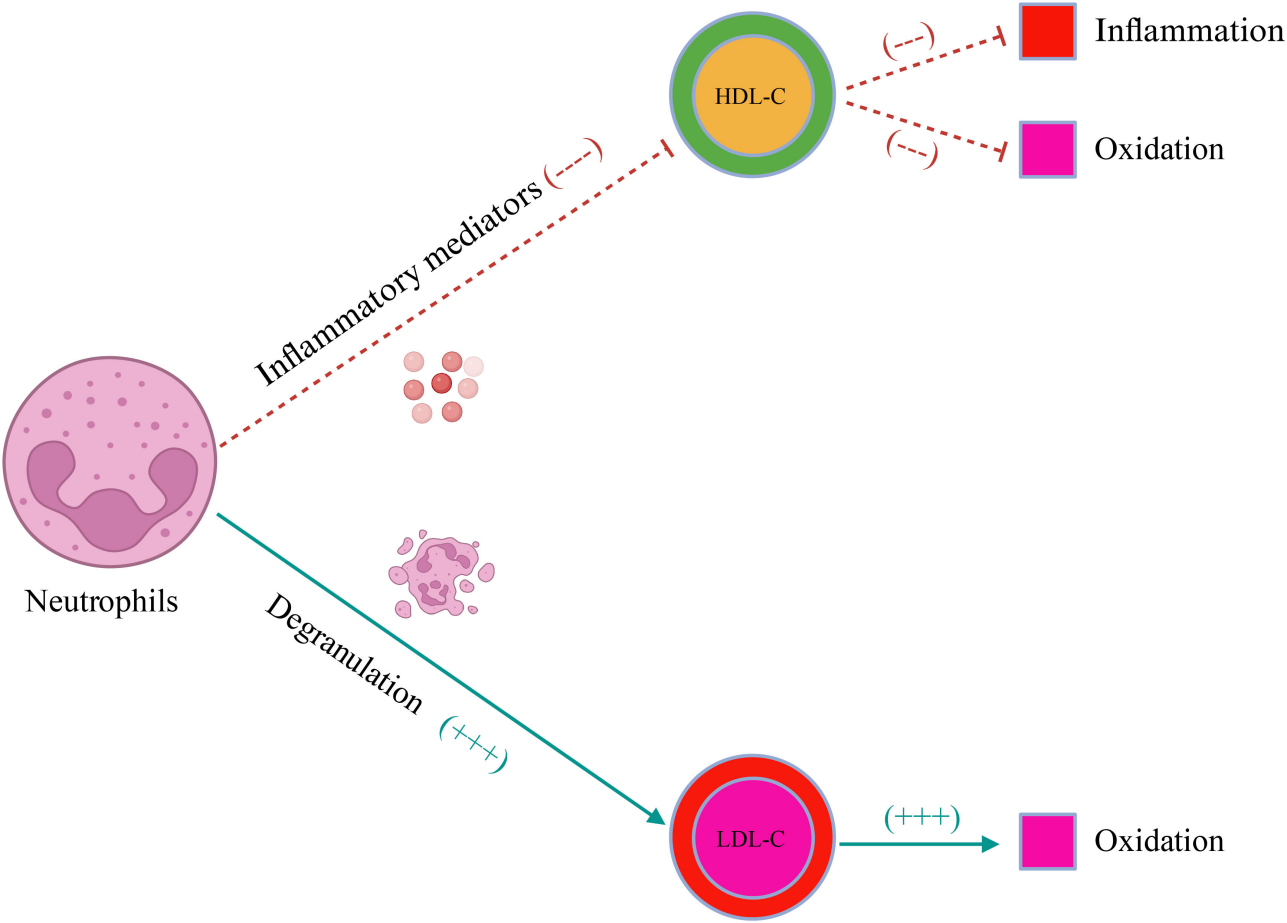
The schematic illustration of the effect of neutrophils on blood lipids. LDL-C, low-density lipoprotein cholesterol; HDL-C, high-density lipoprotein cholesterol.

As a new indicator, NHR reflects the inflammatory state of the body by combining blood cells and blood lipid levels. Neutrophils seem to weaken the anti-oxidant and anti-inflammatory effects of HDL-C, promoting the oxidation of LDL-C by degranulation (**Figure 6**) (29, 30). Although the relationship between NHR and frailty is still unclear, previous studies support that NHR increases the risk of several diseases that may lead to frailty. Studies have shown that NHR is independently associated with the clinical severity of type 2 diabetes mellitus (T2DM), and insulin resistance plays a potential role in the pathogenesis of frailty (31, 32). A study from Qinghai, China shows that NHR is not only an effective predictor of Parkinson’s disease (PD), but also closely related to the disease course. Importantly, Compared with NLR, NHR may be a better predictor of long-term clinical outcomes in PD patients (33). Cohort studies have proven that physical frailty increases the risk of PD by 87% (34). In addition, there is prospective evidence from the UK Biobank that the risk of depression increases in pre-frail and frail people. In the American adult population, an elevated NHR level means a higher risk of depression (14, 35). The evidence mentioned above all supports the potential link between NHR and frailty. However, it should be noted that in the current study, the association between NHR and frailty is J-shaped. When the NHR value is < 3.19, the prevalence of frailty decreases with the increase of NHR. This nonlinear association may be related to the increased risk of cardiovascular events caused by excessively high HDL-C levels. Studies have demonstrated that an excessively high HDL-C concentration may increase the risk of all-cause and CCD-specific deaths (36–39). In people with extremely high HDL-C levels, the probability of atherosclerosis and heart diseases increases, compared with that in people with low HDL-C levels. The concentration of HDL-C in people with partial deletion of the SCARB1 gene is significantly higher and the risk of coronary heart disease is 80% higher than those in normal people (40). There is evidence that CVD and frailty have a bidirectional relationship, and both conditions have several common risk factors and potential biological mechanisms (41).

In terms of the long-term prognosis of frail participants, NHR is positively associated with all-cause, CCD-specific, and cancer-specific mortality risks. According to the study by Shi et al. (42) involving 1,639 patients with hepatocellular carcinoma (HCC) in Beijing, NHR was independently and positively associated with the 3-year mortality rate of HCC patients. Chen et al. (43) found that NHR accurately predicted the short-term mortality rate of HBV-associated decompensated cirrhosis (HBV-DC). In a retrospective cohort study by Li et al. (44) that included 1,051 peritoneal dialysis patients, it was found that NHR was an independent risk factor for all-cause mortality. In 2022, Jiang et al. (15) analyzed the relationship between NHR and survival outcomes in the general population in NHANES. They found that NHR had a U-shaped and linear relationship with all-cause and CVD-specific mortality risks, respectively. The current study further revealed that NHR might serve as a potential marker for the long-term mortality risk in frail individuals.

## Advantages and limitations

The present study has several advantages. First, the sample size is large and the samples are nationally representative since a sampling weight was used in the statistical analysis. Second, the relationship between NHR and the survival outcomes of frailty was also explored. In the cohort, 7,415 frail participants were included with an average follow-up duration of 6.51 years, so the results are relatively reliable. Third, in previous studies on frailty, young participants were often excluded, but participants over 20 years old were included in the present study. Moreover, a robust association between NHR and frailty was detected in both subgroups aged between 20 and 40 and over 40. It further proves the effectiveness of NHR as a frailty marker.

This study also has some limitations. First, it is a cross-sectional study that could not take into account the time sequence. Causal relationships usually require that the cause precedes the effect. However, there is a possibility of reverse causality in the current study, which means frailty may cause certain physiological changes that possibly affect the level of NHR. Although an association between NHR and frailty was observed, it is impossible to determine whether the changes in NHR led to frailty or whether the frailty status affected the level of NHR. To determine the causal relationship between NHR and frailty, future studies with a longitudinal design are required. By conducting a long-term follow-up on the same group of study subjects, the time sequence of the changes in NHR and the occurrence and development of frailty will be observed. It will enable the clarification of the causal relationship between NHR and frailty. Secondly, potential misclassification is an important issue. It is possible that recall bias might lead to misclassification of the frailty status. Similarly, there might also be errors in the measurement of NHR, such as fluctuations in an individual’s physiological state, which might affect the evaluation of the relationship between NHR and frailty. Therefore, there is a need to improve the accuracy and reliability of frailty status assessments and NHR measurements. Multiple measurements can be made on the study subjects to obtain more stable NHR values and frailty status assessments. Finally, it is impossible to completely eliminate the interference of potential confounding factors to the association between NHR and frailty in the present study.

## Conclusion

In the American population aged above 20, NHR shows a J-shaped association with frailty. Moreover, NHR is significantly positively associated with the all-cause, CCD-specific, and cancer-specific mortality risks in frail participants. In conclusion, the present study suggests that NHR may serve as a valuable marker for frailty and its prognosis.

## Data Availability

Publicly available datasets were analyzed in this study. This data can be found here: The website for cross-sectional data is https://wwwn.cdc.gov/nchs/nhanes/; the website for survival data is https://www.cdc.gov/nchs/ndi/index.htm.
