# Supplementary material for: Associations of neutrophil/high-density lipoprotein cholesterol ratio with frailty and its mortality
Source: Front Endocrinol (Lausanne). 2025 Jan 6;15:1495139. doi: 10.3389/fendo.2024.1495139 (PMC11743577; doi:10.3389/fendo.2024.1495139)
Supplement: Supplementary file 1 [file DataSheet1.docx]

**Associations of neutrophil /high-density lipoprotein cholesterol ratio with frailty and its mortality**

Jianqiang Zhang^1,2 MD *^

^1^ Department of Critical Care Medicine, The First Affiliated Hospital, and College of Clinical Medicine of Henan University of Science and Technology, Luoyang, China.

^2^ Department of Neurology, The First Affiliated Hospital, and College of Clinical Medicine of Henan University of Science and Technology, Luoyang, China.

***** Correspondence author: Jianqiang Zhang
E-mail address: jianqiang197901@163.com

Tel: +86 13849909841

## Supplementary Table 1: Variables in Frailty Index and Their Respective Scores

| **Items** | **scores** |
| --- | --- |
| **Cognition** |  |
| 1.experience confusion/memory problems | yes=1, no=0 |
| **Dependence** |  |
| 2.managing money difficulty | no difficulty=0, Some difficulty=0.33, much difficulty=0.66, unable to do=1 |
| 3.walking for a quarter mile difficulty |  |
| 4.walking up ten steps difficulty |  |
| 5.stooping, crouching, kneeling difficulty |  |
| 6.lifting or carrying difficulty |  |
| 7.house chore difficulty |  |
| 8.preparing meals difficulty |  |
| 9.standing up from armless chair difficulty |  |
| 10.getting in and out of bed difficulty |  |
| 11.using fork, knife, drinking from cup difficulty |  |
| 12.dressing yourself difficulty |  |
| 13.standing for long periods difficulty |  |
| 14.grasp/holding small objects difficulty |  |
| 15.attending social event difficulty |  |
| 16.leisure activity at home difficulty |  |
| 17.push or pull large objects difficulty |  |
| **Depressive Conditions** |  |
| 18.have little interest in doing things | nearly every day = 1, more than half the days = 0.66, several days = 0.33, no =0 |
| 19.feeling down, depressed, or hopeless |  |
| 20.trouble sleeping or sleeping too much |  |
| 21.feeling tired or having little energy |  |
| 22.poor appetite or overeating |  |
| 23.feeling bad about yourself |  |
| 24.trouble concentrating on things |  |
| **Comorbidities** |  |
| 25.doctor ever said you had arthritis | yes = 1, no = 0 |
| 26.ever told you had thyroid problem |  |
| 27.ever told you had chronic bronchitis |  |
| 28.ever told you had cancer or malignancy |  |
| 29.ever told had congestive heart failure |  |
| 30.ever told you had coronary heart disease |  |
| 31.ever told you had angina/angina pectoris |  |
| 32.ever told you had heart attack |  |
| 33.ever told you had a stroke |  |
| 34.ever told you had high blood pressure |  |
| 35.doctor told you have diabetes | yes = 1, borderline=0.5, no =0 |
| 36.ever told you had weak/failing kidneys | yes = 1, no =0 |
| 37.urine leakage bother you? | greatly = 1, very much =0.75, somewhat= 0.5, only a little = 0.25, no=0 |
| **Hospital and Care** |  |
| 38.general health condition | excellent, very good, good = 0, fair, poor = 1 |
| 39.health now compared with 1 year ago | worse =1, better = 0 |
| 40.overnight hospital patient in last year | yes =1, no =0 |
| 41.times receive healthcare over past year | no=0, 1-4=0.5, ≥5 =1 |
| 42.number of prescription medicines taken | no =0, 1-4=0.5, ≥5 =1 |
| **Physical Anthropometry** |  |
| 43.body mass index (kg/m^2^) | <18.5, ≥30=1  ≥25, <30=0.5  ≥18.5,＜25=0 |
| **Laboratory values** |  |
| 44.glycohemoglobin (%) | 0%-5.7%=0, >5.7%=1 |
| 45.red blood cell count (million cells/ul) | M: ≥4.7, <6.1=0, Other=1  F: ≥4.2,＜5.4=0, Other =1 |
| 46.hemoglobin (g/dl) | M: ≥13.5, <18 =0, Other =1  F: ≥12, <16 =0, Other=1 |
| 47.red cell distribution width (%) | ≥11.6, <14.6=0, Other=1 |
| 48.lymphocyte percent (%) | ≥20, <40=0, Other=1 |
| 49.segmented neutrophils percent (%) | ≥40, <80=0, Other=1 |

## Supplementary Table 2: Weighted baseline characterization for cohort study

| Characteristics | Total frailty  (N=7,415) | Survivals  (N=5,609) | Death  (N=1,806) | *P*-value |
| --- | --- | --- | --- | --- |
| NHR, Median (IQR) | 3.49(2.38,4.92) | 3.45(2.34,4.86) | 3.60(2.51,5.16) | 0.003 |
| Age(year), Mean (S.E.) | 57.70(0.32) | 54.30(0.32) | 69.83(0.43) | < 0.0001 |
| Total cholesterol level (mmol/L), Mean (S.E.) | 4.93(0.02) | 4.98(0.03) | 4.74(0.03) | < 0.0001 |
| Triglyceride level (mmol/L), Mean (S.E.) | 1.96(0.03) | 1.98(0.04) | 1.87(0.05) | 0.1 |
| Follow-up period (years), Mean (S.E.) | 6.51(0.10) | 6.96(0.13) | 4.91(0.11) | < 0.0001 |
| Low-density lipoprotein cholesterol, Mean (S.E.) | 2.74(2.10,3.44) | 2.82(2.17,3.47) | 2.46(1.91,3.23) | < 0.0001 |
| High-density lipoprotein cholesterol, Mean (S.E.) | 1.27(1.03,1.55) | 1.27(1.03,1.53) | 1.24(1.03,1.58) | 0.96 |
| Castelli risk index I, Mean (S.E.) | 3.75(2.97,4.73) | 3.80(3.00,4.77) | 3.58(2.85,4.59) | < 0.0001 |
| Castelli risk index II, Mean (S.E.) | 2.09(1.55,2.80) | 2.13(1.60,2.86) | 1.93(1.36,2.54) | < 0.0001 |
| Atherogenic index (AI), Mean (S.E.) | 2.74(1.97,3.73) | 2.80(2.00,3.77) | 2.58(1.85,3.59) | < 0.0001 |
| Atherogenic index of plasma (AIP), Mean (S.E.) | 0.03(-0.20, 0.24) | 0.03(-0.20,0.24) | 0.03(-0.19,0.24) | 0.62 |
| Sex, n (%) |  |  |  | < 0.0001 |
| Female | 4,355(62.0) | 3,496(64.2) | 859(53.9) |  |
| Male | 3,060(38.0) | 2,113(35.8) | 947(46.1) |  |
| Race/Ethinicity, n (%) |  |  |  | < 0.0001 |
| Mexican American | 973(6.7) | 829(7.6) | 144(3.4) |  |
| Non-Hispanic Black | 1,905(15.3) | 1,529(16.4) | 376(11.2) |  |
| Non-Hispanic White | 3,244(65.7) | 2,127(62.1) | 1,117(78.7) |  |
| Other Hispanic | 714(5.4) | 623(6.3) | 91(2.0) |  |
| Other Race - Including Multi-Racial | 579(7.1) | 501(7.7) | 78(4.7) |  |
| Educational level, n (%) |  |  |  | < 0.0001 |
| No college | 4,427(52.5) | 3,245(50.6) | 1,182(59.3) |  |
| College or equivalent | 2,988(47.5) | 2,364(49.4) | 624(40.7) |  |
| Marital status, n (%) |  |  |  | < 0.0001 |
| No married | 907(11.3) | 793(12.8) | 114(6.0) |  |
| Divorced or separated or widowed | 2,741(33.4) | 1,864(29.7) | 877(46.8) |  |
| Already married or cohabitation | 3,762(55.2) | 2,949(57.5) | 813(47.3) |  |
| PIR, n (%) |  |  |  | < 0.0001 |
| <1.3 | 2,965(31.5) | 2,273(31.5) | 692(31.6) |  |
| 1.3–3.5 | 2,559(36.6) | 1,849(35.1) | 710(41.8) |  |
| >3.5 | 1,180(24.2) | 939(25.8) | 241(18.3) |  |
| Not report | 711(7.8) | 548(7.6) | 163(8.2) |  |
| Drinking status, n (%) |  |  |  | < 0.0001 |
| Never drinked | 1,038(11.2) | 744(10.0) | 294(15.6) |  |
| Former drinker | 1,774(22.0) | 1,101(18.4) | 673(35.1) |  |
| Current drinker | 3,638(54.9) | 2,999(59.4) | 639(39.1) |  |
| Not report | 965(11.9) | 765(12.3) | 200(10.3) |  |
| Smoking status, n (%) |  |  |  | < 0.0001 |
| Never smoked | 3,249(42.1) | 2,568(43.5) | 681(37.2) |  |
| Former smoker | 2,328(31.2) | 1,592(28.8) | 736(39.9) |  |
| Current smoker | 1,838(26.7) | 1,449(27.8) | 389(22.9) |  |
| Physical activity (MET, minutes/week, n (%) |  |  |  | < 0.0001 |
| <700 | 1,391(19.3) | 1,049(19.5) | 342(18.6) |  |
| 700-2400 | 1,206(17.3) | 991(18.4) | 215(13.3) |  |
| >=2400 | 1,508(22.1) | 1,359(25.9) | 149(8.6) |  |
| Not report | 3,310(41.3) | 2,210(36.2) | 1,100(59.5) |  |
| Energy intake (kcal/day), n (%) |  |  |  | < 0.0001 |
| Low | 3,689(47.4) | 2,719(45.8) | 970(53.1) |  |
| High | 3,164(45.9) | 2,485(47.8) | 679(38.8) |  |
| Not report | 562(6.8) | 405(6.4) | 157(8.1) |  |
| Body mass index, n (%) |  |  |  | < 0.0001 |
| <25 kg/m^2^ | 1,429(18.8) | 918(16.5) | 511(27.3) |  |
| >=25 kg/m^2^ | 5,986(81.2) | 4691(83.5) | 1,295(72.7) |  |
| Statins use, n (%) |  |  |  | < 0.0001 |
| No | 4,521(62.5) | 3,563(65.0) | 958(53.5) |  |
| Yes | 2,894(37.5) | 2,046(35.0) | 848(46.5) |  |
| Cancer, n (%) |  |  |  | < 0.0001 |
| No | 6,040(79.2) | 4,728(81.4) | 1,312(71.5) |  |
| Yes | 1,375(20.8) | 881(18.6) | 494(28.5) |  |
| DM, n (%) |  |  |  | < 0.0001 |
| DM | 3,180(38.1) | 2,293(36.1) | 887(45.1) |  |
| IFG | 354(5.6) | 267(5.5) | 87(6.2) |  |
| IGT | 251(3.4) | 172(3.1) | 79(4.5) |  |
| No | 3,630(52.9) | 2,877(55.4) | 753(44.3) |  |
| CVD, n (%) |  |  |  | < 0.0001 |
| No | 4,868(68.2) | 4,012(73.5) | 856(49.5) |  |
| Yes | 2,547(31.8) | 1,597(26.5) | 950(50.5) |  |
| Hypertension, n (%) |  |  |  | < 0.0001 |
| No | 1,996(29.3) | 1,677(32.4) | 319(18.2) |  |
| Yes | 5,419(70.7) | 3,932(67.6) | 1,487(81.8) |  |
| Statins categories, n (%) |  |  |  | < 0.0001 |
| Low intensity | 5(0.2) | 5(0.3) | 0(0.0) |  |
| Moderate intensity | 1,631(21.2) | 1,116(19.4) | 515(28.1) |  |
| High intensity | 1,255(16.0) | 924(15.3) | 331(18.4) |  |
| Not use | 4,519(62.5) | 3,561(65.0) | 958(53.5) |  |
| Statins combine with Ezetimibe, n (%) |  |  |  | < 0.001 |
| No | 7,244(97.7) | 5,504(98.2) | 1,740(96.3) |  |
| Yes | 166(2.2) | 102(1.8) | 64(3.7) |  |
| Fibrates use, n (%) |  |  |  | 0.42 |
| No | 7,242(97.4) | 5,491(97.6) | 1,751(97.1) |  |
| Yes | 168(2.6) | 115(2.4) | 53(2.9) |  |

Notes: NHR, neutrophil/high-density lipoprotein cholesterol ratio; IQR, Interquartile Range; SE, standard error; PIR, poverty-to-income ratio; MET, metabolic equivalent; DM, diabetes mellitus; IFG, impaired fasting glycaemia; IGT, impaired glucose tolerance; CVD, cardiovascular diseases.

## Supplementary Table 3: The association of NHR with vulnerable/ frail/ most frail (Sensitivity analysis 1).

|  |  | Model 0 | | Model 1 | | Model 2 | | Model 3 | | |
| --- | --- | --- | --- | --- | --- | --- | --- | --- | --- | --- |
|  |  | OR (95%CI) | *P-*value | OR (95%CI) | *P-*value | OR (95%CI) | *P-*value | OR (95%CI) | *P-*value |  |
| Vulnerable/Non-frail ~NHR | Per SD | 1.08(1.04,1.12) | <0.001 | 1.23(1.18,1.28) | <0.0001 | 1.11(1.07,1.16) | <0.0001 | 1.05(1.01,1.10) | 0.03 |  |
| Frail/Non-frail ~NHR | Per SD | 1.39(1.32,1.47) | <0.0001 | 1.75(1.63, 1.87) | <0.0001 | 1.45(1.36, 1.55) | <0.0001 | 1.12(1.03, 1.23) | 0.01 |  |
| Most frail/Non-frail ~NHR | Per SD | 1.66(1.51,1.82) | <0.0001 | 2.08(1.79,2.43) | <0.0001 | 1.61(1.37,1.89) | <0.0001 | 1.31(1.08, 1.60) | 0.01 |  |

Notes: Model 0: Crude model. Model 1: Adjusted for age, sex, race, marital status, education, and poverty-income ratio. Model 2: Additionally adjusted for drinking, smoking, total energy intake, weekly physical activity level, and BMI. Model 3: Additionally, adjusted for diabetes, cancer, hypertension, CVD, blood cholesterol levels, blood triglyceride levels, and statins. NHR, neutrophil/high-density lipoprotein cholesterol ratio; SD, standard deviation; BMI, body mass index; OR, odds ratio; CI, confidence interval.

## Supplementary Table 4: Further adjust for categorical statins, statins combine with Ezetimibe, and fibrates (Sensitivity analysis 2).

|  |  | Model 0 | | Model 1 | | Model 2 | | Model 3 | |
| --- | --- | --- | --- | --- | --- | --- | --- | --- | --- |
|  |  | OR (95%CI) | *P-*value | OR (95%CI) | *P-*value | OR (95%CI) | *P-*value | OR (95%CI) | *P-*value |
| Frailty ~NHR | Per SD | 1.34(1.28,1.40) | <0.0001 | 1.53(1.45, 1.61) | <0.0001 | 1.34(1.27, 1.41) | <0.0001 | 1.11(1.04, 1.19) | 0.001 |
|  |  |  |  |  |  |  |  |  |  |
|  |  | HR (95%CI) | *P-*value | HR (95%CI) | *P-*value | HR (95%CI) | *P-*value | HR (95%CI) | *P-*value |
| All-cause mortality ~NHR | Per SD | 1.12(1.07,1.18) | <0.0001 | 1.15(1.10,1.20) | <0.0001 | 1.13(1.08,1.18) | <0.0001 | 1.12(1.07,1.18) | <0.0001 |
| CVD mortality ~NHR | Per SD | 1.12(1.04,1.20) | 0.002 | 1.24(1.14,1.34) | <0.0001 | 1.24(1.13,1.35) | <0.0001 | 1.22(1.11, 1.34) | <0.0001 |
| Cancer mortality ~NHR | Per SD | 1.17(1.05,1.30) | 0.004 | 1.15(1.10, 1.21) | <0.0001 | 1.12(1.06, 1.18) | <0.0001 | 1.13(1.07, 1.19) | <0.0001 |

Notes: Model 0: Crude model. Model 1: Adjusted for age, sex, race, marital status, education, and poverty-income ratio. Model 2: Additionally adjusted for drinking, smoking, total energy intake, weekly physical activity level, and BMI. Model 3: Additionally, adjusted for diabetes, cancer, hypertension, CVD, blood cholesterol levels, blood triglyceride levels, Categorical statins, **Statins combine with Ezetimibe, and fibrates**. NHR, neutrophil/high-density lipoprotein cholesterol ratio; SD, standard deviation; BMI, body mass index; OR, odds ratio; HR, hazard ratio; CI, confidence interval.

## Supplementary Table 5: NHR was divided into two categories (high and low) based on the median value for regression analysis (Sensitivity analysis 3).

|  | Model 0 | | Model 1 | | Model 2 | | Model 3 | |
| --- | --- | --- | --- | --- | --- | --- | --- | --- |
|  | HR (95%CI) | *P-*value | HR (95%CI) | *P-*value | HR (95%CI) | *P-*value | HR (95%CI) | *P-*value |
| All-cause mortality ~Dichotomous NHR | 1.17(1.04,1.32) | 0.01 | 1.25(1.09,1.42) | 0.001 | 1.22(1.06,1.40) | 0.004 | 1.17(1.01, 1.36) | 0.04 |
| CVD mortality ~ Dichotomous NHR | 1.33(1.07,1.65) | 0.01 | 1.46(1.17,1.82) | <0.001 | 1.46(1.16,1.84) | 0.001 | 1.43(1.11,1.84) | 0.01 |
| Cancer mortality ~ Dichotomous NHR | 1.06(0.80,1.41) | 0.67 | 1.10(0.80, 1.50) | 0.56 | 1.05(0.77, 1.45) | 0.74 | 1.07(0.75, 1.51) | 0.71 |

Notes: Model 0: Crude model. Model 1: Adjusted for age, sex, race, marital status, education, and poverty-income ratio. Model 2: Additionally adjusted for drinking, smoking, total energy intake, weekly physical activity level, and BMI. Model 3: Additionally, adjusted for diabetes, cancer, hypertension, CVD, blood cholesterol levels, blood triglyceride levels, and statins. NHR, neutrophil/high-density lipoprotein cholesterol ratio; SD, standard deviation; BMI, body mass index; HR, hazard ratio; CI, confidence interval.

## Supplementary Figure 1: Kaplan Meier curves of cumulative risk for mortality between low group and high group of NHR.


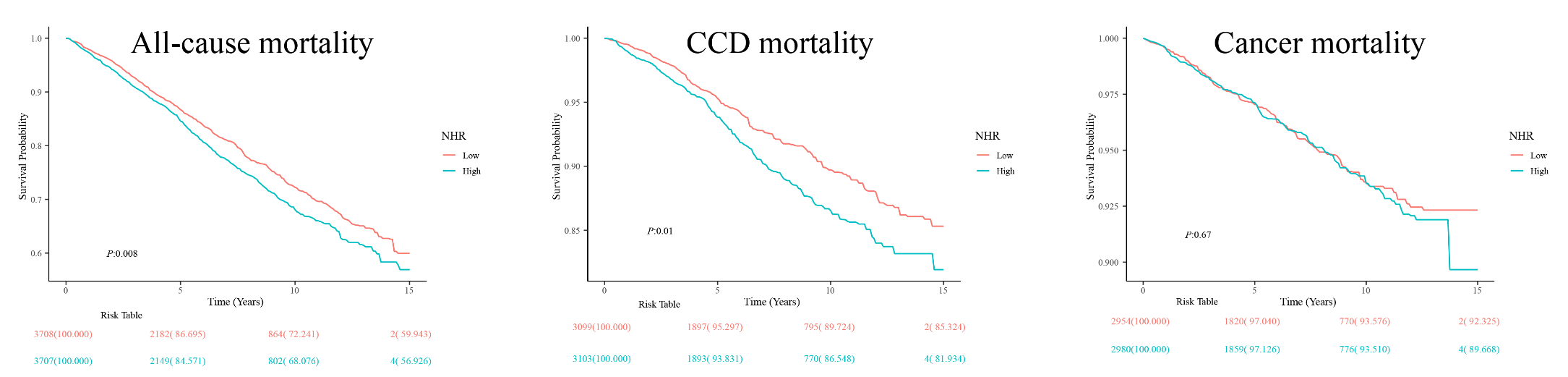


Notes: NHR, neutrophil/high-density lipoprotein cholesterol ratio; CCD, cardiocerebrovascular disease.
